# (*Z*)-Ethyl 2-cyano-2-{2-[5,6-dimethyl-4-(thio­phen-2-yl)-1*H*-pyrazolo­[3,4-*b*]pyridin-3-yl]hydrazinylidene}acetate

**DOI:** 10.1107/S1600536811028911

**Published:** 2011-07-30

**Authors:** Hoong-Kun Fun, Madhukar Hemamalini, Hatem A. Abdel-Aziz, Tarek Aboul-Fadl

**Affiliations:** a X-ray Crystallography Unit, School of Physics, Universiti Sains Malaysia, 11800 USM, Penang, Malaysia; b Department of Pharmaceutical Chemistry, College of Pharmacy, King Saud University, Riyadh 11451, Saudi Arabia

## Abstract

In the title compound, C_17_H_16_N_6_O_2_S, an intra­molecular N—H⋯O inter­action generates an *S*(6) ring. The pyridine ring makes a dihedral angle of 71.38 (11)° with the thio­phene ring. In the crystal, mol­ecules are linked by a pair of N—H⋯N hydrogen bonds, forming an inversion dimer. The dimers are stacked in columns along the *b* axis through weak inter­molecular C—H⋯N hydrogen bonds.

## Related literature

For applications of pyrazole derivatives, see: Casas *et al.* (2007 ▶); Habeeb *et al.* (2001 ▶); Hashimoto *et al.* (2002 ▶); Ranatunge *et al.* (2004 ▶); Singh *et al.* (2005 ▶); Elzein *et al.* (2006 ▶). For previous reports on the diazotization of heterocyclic amines, see: Abdel-Aziz *et al.* (2008 ▶); Hamdy *et al.* (2007 ▶); Dawood *et al.* (2005 ▶); Farag *et al.* (2004 ▶). For graph-set notation, see: Bernstein *et al.* (1995 ▶).
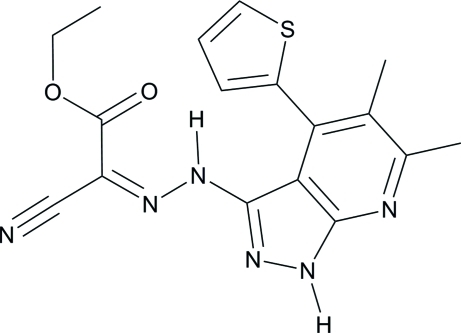


## Experimental

### 

#### Crystal data

C_17_H_16_N_6_O_2_S
*M* *_r_* = 368.42
Monoclinic, 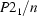

*a* = 8.0672 (5) Å
*b* = 10.6460 (7) Å
*c* = 20.5892 (13) Å
β = 90.332 (1)°
*V* = 1768.24 (19) Å^3^
*Z* = 4
Mo *K*α radiation
μ = 0.21 mm^−1^
*T* = 296 K
0.47 × 0.22 × 0.13 mm

#### Data collection

Bruker APEXII DUO CCD area-detector diffractometer
Absorption correction: multi-scan (*SADABS*; Bruker, 2009 ▶) *T*_min_ = 0.908, *T*_max_ = 0.974
33610 measured reflections
4782 independent reflections
3518 reflections with *I* > 2σ(*I*)
*R*_int_ = 0.029

#### Refinement

*R*[*F* ^2^ > 2σ(*F* ^2^)] = 0.063
*wR*(*F* ^2^) = 0.219
*S* = 1.06
4782 reflections
238 parameters
H-atom parameters constrained
Δρ_max_ = 0.61 e Å^−3^
Δρ_min_ = −0.68 e Å^−3^

### 

Data collection: *APEX2* (Bruker, 2009 ▶); cell refinement: *SAINT* (Bruker, 2009 ▶); data reduction: *SAINT*; program(s) used to solve structure: *SHELXTL* (Sheldrick, 2008 ▶); program(s) used to refine structure: *SHELXTL*; molecular graphics: *SHELXTL*; software used to prepare material for publication: *SHELXTL* and *PLATON* (Spek, 2009 ▶).

## Supplementary Material

Crystal structure: contains datablock(s) global, I. DOI: 10.1107/S1600536811028911/is2753sup1.cif

Structure factors: contains datablock(s) I. DOI: 10.1107/S1600536811028911/is2753Isup2.hkl

Supplementary material file. DOI: 10.1107/S1600536811028911/is2753Isup3.cml

Additional supplementary materials:  crystallographic information; 3D view; checkCIF report

## Figures and Tables

**Table 1:** Hydrogen-bond geometry (Å, °)

*D*—H⋯*A*	*D*—H	H⋯*A*	*D*⋯*A*	*D*—H⋯*A*
N2—H1*N*2⋯N1^i^	0.89	2.07	2.941 (3)	165
N4—H1*N*4⋯O1	0.85	1.99	2.642 (3)	133
C13—H13*A*⋯N5^ii^	0.93	2.62	3.463 (3)	151

Symmetry codes: (i) 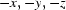; (ii) 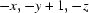.

**Fig. 1.**
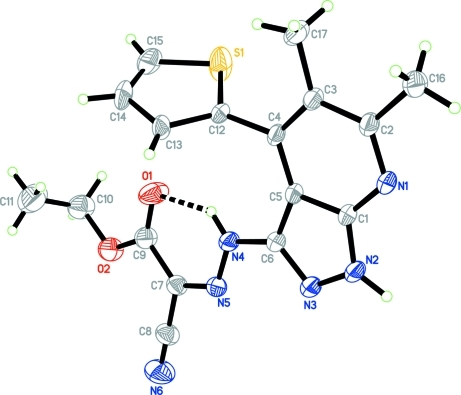
The asymmetric unit of the title compound, showing 30% probability displacement ellipsoids and the atom-numbering scheme. The intramolecular hydrogen bond is shown by a dashed line.

**Fig. 2.**
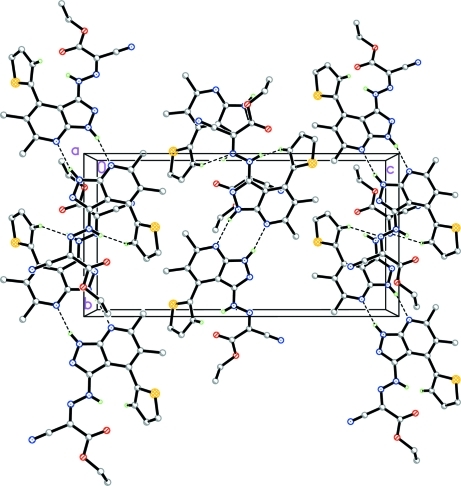
The crystal packing of the title compound. Dashed lines represent hydrogen bonding.

## supplementary crystallographic information

### Comment

Pyrazolones form a very important class of heterocycles due to their properties and applications (Casas *et al.*, 2007). The pyrazole unit is one of the core structures in a number of natural products and has attracted attention in the field of biological sciences (Habeeb *et al.*, 2001; Hashimoto *et al.*, 2002). Extensive studies have been devoted to arylpyrazole derivatives such as celecoxib, a well-known cyclooxygenase-2 inhibitor (Ranatunge *et al.*, 2004; Singh *et al.*, 2005). Recently, pyrazole derivatives have been reported as high affinity and selective A2B adenosine receptor antagonist (Elzein *et al.*, 2006). In continuation of our studies on pyrazolone schiff bases, we herein report the crystal structure of the title pyrazole compound.

The molecular structure of the title compound is shown in Fig. 1. The pyridine ring (N1/C1–C5) is essentially planar [maximum deivation of 0.010 (2) Å for atom C5] and makes dihedral angles of 71.38 (11)° and 1.92 (12)° with the thiophene (S1/C12–C15) and pyrazole (N2/N3/C1/C5/C6) rings.

The crystal structure, (Fig. 2), is stabilized by weak intermolecular N2—H1N2···N1 and C13—H13A···N5  (Table 1) hydrogen bonds. There is an intramolecular N4–H1N4···O1 interaction generating an *S*(6) ring (Bernstein *et al.*, 1995).

### Experimental

To a stirred solution of ethyl cyanoacetate (1.3 g, 10 mmol) in ethanol (50 ml), sodium acetate trihydrate (1.3 g, 10 mmol) was added. After stirring for 15 min, the mixture was chilled at 5°C and treated with a cold solution of 5,6-dimethyl-4-(thiophen-2-yl)-1*H*-pyrazolo[3,4-b]pyridin-3-amine dizonium chloride  with stirring for 2 h at 0–5 °C. The mixture was then left for 8h in a refrigerator (4 °C). The resulting solid was collected by filtration, washed thoroughly with water and dried. The crude product was crystallized from ethanol to give the title hydrazone.

Preparation of 5,6-dimethyl-4-(thiophen-2-yl)-1*H*-pyrazolo[3,4-b] pyridin-3-amine dizonium chloride.

A suspension of 5,6-dimethyl-4-(thiophen-2-yl)-1*H*-pyrazolo[3,4-b]pyridin-3-amine (2.44 g, 10 mmol) in glacial acetic acid (10 ml) was heated to produce a clear solution and then cooled to 5 °C. 15 ml of hydrochloric acid was then added. A solution of sodium nitrite (0.7 g, 10 mmol) in water (10 ml) was then gradually added with stirring.

### Refinement

All hydrogen atoms were positioned geometrically (N—H = 0.8475–0.8953 Å; C—H = 0.93–0.97 Å) and were refined using a riding model, with *U*_iso_(H) = 1.2 or 1.5*U*_eq_(C). A rotating group model was applied to the methyl groups.

### Crystal data

**Table:**

C_17_H_16_N_6_O_2_S	*F*(000) = 768
*M**_r_* = 368.42	*D*_x_ = 1.384 Mg m^−^^3^
Monoclinic, *P*2_1_/*n*	Mo *K*α radiation, λ = 0.71073 Å
Hall symbol: -P 2yn	Cell parameters from 8773 reflections
*a* = 8.0672 (5) Å	θ = 2.7–28.9°
*b* = 10.6460 (7) Å	µ = 0.21 mm^−^^1^
*c* = 20.5892 (13) Å	*T* = 296 K
β = 90.332 (1)°	Block, yellow
*V* = 1768.24 (19) Å^3^	0.47 × 0.22 × 0.13 mm
*Z* = 4	

### Data collection

**Table:**

Bruker APEXII DUO CCD area-detector diffractometer	4782 independent reflections
Radiation source: fine-focus sealed tube	3518 reflections with *I* > 2σ(*I*)
graphite	*R*_int_ = 0.029
φ and ω scans	θ_max_ = 29.2°, θ_min_ = 2.0°
Absorption correction: multi-scan (*SADABS*; Bruker, 2009)	*h* = −11→11
*T*_min_ = 0.908, *T*_max_ = 0.974	*k* = −14→14
33610 measured reflections	*l* = −28→28

### Refinement

**Table:**

Refinement on *F*^2^	Primary atom site location: structure-invariant direct methods
Least-squares matrix: full	Secondary atom site location: difference Fourier map
*R*[*F*^2^ > 2σ(*F*^2^)] = 0.063	Hydrogen site location: inferred from neighbouring sites
*wR*(*F*^2^) = 0.219	H-atom parameters constrained
*S* = 1.06	*w* = 1/[σ^2^(*F*_o_^2^) + (0.1182*P*)^2^ + 0.809*P*] where *P* = (*F*_o_^2^ + 2*F*_c_^2^)/3
4782 reflections	(Δ/σ)_max_ = 0.001
238 parameters	Δρ_max_ = 0.61 e Å^−^^3^
0 restraints	Δρ_min_ = −0.68 e Å^−^^3^

### Special details

**Table:**

Geometry. All s.u.'s (except the s.u. in the dihedral angle between two l.s. planes) are estimated using the full covariance matrix. The cell s.u.'s are taken into account individually in the estimation of s.u.'s in distances, angles and torsion angles; correlations between s.u.'s in cell parameters are only used when they are defined by crystal symmetry. An approximate (isotropic) treatment of cell s.u.'s is used for estimating s.u.'s involving l.s. planes.
Refinement. Refinement of F^2^ against ALL reflections. The weighted R-factor wR and goodness of fit S are based on F^2^, conventional R-factors R are based on F, with F set to zero for negative F^2^. The threshold expression of F^2^ > 2σ(F^2^) is used only for calculating R-factors(gt) etc. and is not relevant to the choice of reflections for refinement. R-factors based on F^2^ are statistically about twice as large as those based on F, and R- factors based on ALL data will be even larger.

### Fractional atomic coordinates and isotropic or equivalent isotropic displacement parameters (Å^2^)

**Table:**

	*x*	*y*	*z*	*U*_iso_*/*U*_eq_	
O1	0.3443 (3)	0.6604 (2)	0.08593 (9)	0.0832 (7)	
O2	0.4554 (3)	0.80464 (18)	0.01904 (9)	0.0674 (5)	
N1	−0.0513 (2)	0.07181 (16)	0.08467 (9)	0.0443 (4)	
N2	0.0682 (3)	0.16420 (17)	−0.01066 (9)	0.0527 (5)	
H1N2	0.0709	0.1009	−0.0392	0.063*	
N3	0.1472 (3)	0.27295 (17)	−0.02803 (9)	0.0514 (5)	
N4	0.2184 (2)	0.46227 (16)	0.02614 (8)	0.0447 (4)	
H1N4	0.2299	0.5014	0.0617	0.054*	
N5	0.2772 (2)	0.51079 (17)	−0.02755 (8)	0.0441 (4)	
N6	0.4563 (4)	0.7030 (3)	−0.13610 (14)	0.0921 (9)	
C1	0.0215 (3)	0.16591 (18)	0.05219 (9)	0.0413 (4)	
C2	−0.0843 (3)	0.09562 (19)	0.14668 (10)	0.0418 (4)	
C3	−0.0472 (2)	0.21163 (18)	0.17806 (10)	0.0407 (4)	
C4	0.0297 (2)	0.30706 (17)	0.14358 (9)	0.0370 (4)	
C5	0.0674 (2)	0.28297 (17)	0.07806 (9)	0.0375 (4)	
C6	0.1458 (3)	0.34311 (19)	0.02462 (9)	0.0419 (4)	
C7	0.3502 (3)	0.6208 (2)	−0.02605 (10)	0.0465 (5)	
C8	0.4084 (3)	0.6659 (3)	−0.08754 (13)	0.0588 (6)	
C9	0.3807 (3)	0.6957 (2)	0.03246 (12)	0.0554 (6)	
C10	0.4980 (4)	0.8842 (3)	0.07369 (16)	0.0752 (8)	
H10A	0.5939	0.9350	0.0631	0.090*	
H10B	0.5258	0.8326	0.1110	0.090*	
C11	0.3568 (6)	0.9662 (4)	0.0894 (2)	0.1057 (13)	
H11A	0.3878	1.0224	0.1238	0.158*	
H11B	0.2646	0.9158	0.1029	0.158*	
H11C	0.3261	1.0140	0.0516	0.158*	
C12	0.0656 (2)	0.43206 (17)	0.17233 (9)	0.0385 (4)	
S1	0.21437 (12)	0.45313 (7)	0.23040 (5)	0.0874 (4)	
C13	−0.0232 (3)	0.54559 (17)	0.16053 (11)	0.0471 (5)	
H13A	−0.1118	0.5552	0.1319	0.056*	
C14	0.0476 (3)	0.6427 (2)	0.20046 (14)	0.0605 (7)	
H14A	0.0110	0.7255	0.1989	0.073*	
C15	0.1673 (4)	0.6062 (3)	0.23924 (14)	0.0674 (7)	
H15A	0.2207	0.6591	0.2686	0.081*	
C16	−0.1660 (4)	−0.0089 (2)	0.18327 (13)	0.0591 (6)	
H16A	−0.1719	−0.0823	0.1563	0.089*	
H16B	−0.2759	0.0161	0.1954	0.089*	
H16C	−0.1024	−0.0275	0.2217	0.089*	
C17	−0.1005 (4)	0.2290 (2)	0.24741 (12)	0.0590 (6)	
H17A	−0.0752	0.3130	0.2612	0.089*	
H17B	−0.0426	0.1702	0.2746	0.089*	
H17C	−0.2177	0.2149	0.2506	0.089*	

### Atomic displacement parameters (Å^2^)

**Table:**

	*U*^11^	*U*^22^	*U*^33^	*U*^12^	*U*^13^	*U*^23^
O1	0.1284 (19)	0.0709 (13)	0.0503 (10)	−0.0409 (13)	0.0105 (11)	−0.0096 (9)
O2	0.0854 (13)	0.0548 (10)	0.0619 (11)	−0.0234 (9)	−0.0050 (9)	0.0013 (8)
N1	0.0605 (10)	0.0309 (8)	0.0415 (9)	−0.0040 (7)	−0.0020 (7)	−0.0093 (6)
N2	0.0854 (14)	0.0356 (9)	0.0372 (9)	−0.0096 (9)	0.0032 (9)	−0.0098 (7)
N3	0.0774 (13)	0.0385 (9)	0.0384 (9)	−0.0054 (9)	0.0012 (8)	−0.0044 (7)
N4	0.0599 (11)	0.0372 (8)	0.0371 (8)	−0.0072 (7)	0.0009 (7)	−0.0031 (6)
N5	0.0515 (10)	0.0407 (9)	0.0401 (9)	−0.0024 (7)	−0.0033 (7)	0.0010 (7)
N6	0.120 (2)	0.094 (2)	0.0626 (15)	−0.0126 (17)	0.0142 (15)	0.0220 (14)
C1	0.0559 (11)	0.0300 (9)	0.0381 (9)	−0.0006 (8)	−0.0031 (8)	−0.0071 (7)
C2	0.0488 (10)	0.0325 (9)	0.0441 (10)	−0.0020 (8)	0.0013 (8)	−0.0064 (7)
C3	0.0467 (10)	0.0355 (9)	0.0400 (9)	0.0001 (8)	0.0033 (8)	−0.0090 (7)
C4	0.0420 (9)	0.0296 (8)	0.0394 (9)	0.0022 (7)	−0.0028 (7)	−0.0094 (7)
C5	0.0460 (10)	0.0285 (8)	0.0379 (9)	0.0012 (7)	−0.0033 (7)	−0.0060 (7)
C6	0.0539 (11)	0.0341 (9)	0.0375 (9)	−0.0010 (8)	−0.0024 (8)	−0.0036 (7)
C7	0.0519 (11)	0.0434 (11)	0.0443 (11)	−0.0040 (9)	−0.0033 (9)	0.0029 (8)
C8	0.0656 (15)	0.0572 (14)	0.0536 (13)	−0.0080 (12)	−0.0014 (11)	0.0084 (11)
C9	0.0665 (14)	0.0486 (12)	0.0512 (12)	−0.0124 (11)	0.0006 (10)	−0.0013 (10)
C10	0.088 (2)	0.0700 (18)	0.0674 (17)	−0.0241 (16)	−0.0069 (15)	−0.0054 (14)
C11	0.123 (3)	0.104 (3)	0.090 (3)	0.005 (3)	0.022 (2)	−0.018 (2)
C12	0.0471 (10)	0.0322 (9)	0.0362 (9)	−0.0007 (7)	−0.0024 (7)	−0.0092 (7)
S1	0.1022 (6)	0.0517 (4)	0.1075 (7)	0.0073 (4)	−0.0565 (5)	−0.0211 (4)
C13	0.0628 (13)	0.0254 (8)	0.0529 (12)	−0.0027 (8)	−0.0138 (10)	−0.0069 (8)
C14	0.0720 (16)	0.0301 (10)	0.0795 (17)	0.0007 (10)	0.0077 (13)	−0.0153 (10)
C15	0.0914 (19)	0.0490 (13)	0.0617 (15)	−0.0171 (13)	−0.0097 (14)	−0.0215 (11)
C16	0.0800 (16)	0.0428 (11)	0.0544 (13)	−0.0172 (11)	0.0066 (12)	−0.0038 (10)
C17	0.0762 (16)	0.0506 (13)	0.0504 (12)	−0.0084 (12)	0.0185 (11)	−0.0156 (10)

### Geometric parameters (Å, °)

**Table:**

O1—C9	1.202 (3)	C7—C8	1.436 (3)
O2—C9	1.337 (3)	C7—C9	1.464 (3)
O2—C10	1.448 (3)	C10—C11	1.472 (5)
N1—C2	1.330 (3)	C10—H10A	0.9700
N1—C1	1.342 (3)	C10—H10B	0.9700
N2—C1	1.350 (3)	C11—H11A	0.9600
N2—N3	1.370 (3)	C11—H11B	0.9600
N2—H1N2	0.8953	C11—H11C	0.9600
N3—C6	1.317 (3)	C12—C13	1.425 (3)
N4—N5	1.312 (2)	C12—S1	1.704 (2)
N4—C6	1.398 (3)	S1—C15	1.683 (3)
N4—H1N4	0.8475	C13—C14	1.438 (3)
N5—C7	1.311 (3)	C13—H13A	0.9300
N6—C8	1.144 (3)	C14—C15	1.309 (4)
C1—C5	1.404 (2)	C14—H14A	0.9300
C2—C3	1.425 (3)	C15—H15A	0.9300
C2—C16	1.498 (3)	C16—H16A	0.9600
C3—C4	1.388 (3)	C16—H16B	0.9600
C3—C17	1.505 (3)	C16—H16C	0.9600
C4—C5	1.408 (3)	C17—H17A	0.9600
C4—C12	1.484 (2)	C17—H17B	0.9600
C5—C6	1.424 (3)	C17—H17C	0.9600
			
C9—O2—C10	116.9 (2)	O2—C10—H10A	109.7
C2—N1—C1	115.27 (17)	C11—C10—H10A	109.7
C1—N2—N3	111.82 (16)	O2—C10—H10B	109.7
C1—N2—H1N2	130.4	C11—C10—H10B	109.7
N3—N2—H1N2	116.9	H10A—C10—H10B	108.2
C6—N3—N2	104.96 (17)	C10—C11—H11A	109.5
N5—N4—C6	119.50 (17)	C10—C11—H11B	109.5
N5—N4—H1N4	119.8	H11A—C11—H11B	109.5
C6—N4—H1N4	120.6	C10—C11—H11C	109.5
C7—N5—N4	119.77 (18)	H11A—C11—H11C	109.5
N1—C1—N2	126.38 (17)	H11B—C11—H11C	109.5
N1—C1—C5	126.08 (18)	C13—C12—C4	126.55 (17)
N2—C1—C5	107.51 (18)	C13—C12—S1	111.06 (14)
N1—C2—C3	123.90 (19)	C4—C12—S1	122.20 (15)
N1—C2—C16	115.61 (18)	C15—S1—C12	92.58 (12)
C3—C2—C16	120.49 (19)	C12—C13—C14	108.4 (2)
C4—C3—C2	119.73 (18)	C12—C13—H13A	125.8
C4—C3—C17	121.76 (18)	C14—C13—H13A	125.8
C2—C3—C17	118.46 (19)	C15—C14—C13	115.1 (2)
C3—C4—C5	117.16 (16)	C15—C14—H14A	122.5
C3—C4—C12	122.62 (17)	C13—C14—H14A	122.5
C5—C4—C12	120.17 (17)	C14—C15—S1	112.84 (18)
C1—C5—C4	117.84 (18)	C14—C15—H15A	123.6
C1—C5—C6	102.90 (16)	S1—C15—H15A	123.6
C4—C5—C6	139.26 (18)	C2—C16—H16A	109.5
N3—C6—N4	121.85 (19)	C2—C16—H16B	109.5
N3—C6—C5	112.78 (18)	H16A—C16—H16B	109.5
N4—C6—C5	125.35 (17)	C2—C16—H16C	109.5
N5—C7—C8	115.3 (2)	H16A—C16—H16C	109.5
N5—C7—C9	125.4 (2)	H16B—C16—H16C	109.5
C8—C7—C9	119.3 (2)	C3—C17—H17A	109.5
N6—C8—C7	179.0 (3)	C3—C17—H17B	109.5
O1—C9—O2	125.0 (2)	H17A—C17—H17B	109.5
O1—C9—C7	122.8 (2)	C3—C17—H17C	109.5
O2—C9—C7	112.1 (2)	H17A—C17—H17C	109.5
O2—C10—C11	109.7 (3)	H17B—C17—H17C	109.5
			
C1—N2—N3—C6	−1.2 (3)	N5—N4—C6—N3	6.0 (3)
C6—N4—N5—C7	−177.88 (19)	N5—N4—C6—C5	−175.59 (19)
C2—N1—C1—N2	179.4 (2)	C1—C5—C6—N3	0.5 (2)
C2—N1—C1—C5	1.5 (3)	C4—C5—C6—N3	179.5 (2)
N3—N2—C1—N1	−176.7 (2)	C1—C5—C6—N4	−178.0 (2)
N3—N2—C1—C5	1.6 (3)	C4—C5—C6—N4	1.0 (4)
C1—N1—C2—C3	0.1 (3)	N4—N5—C7—C8	−179.3 (2)
C1—N1—C2—C16	179.9 (2)	N4—N5—C7—C9	3.1 (4)
N1—C2—C3—C4	−0.8 (3)	C10—O2—C9—O1	1.3 (4)
C16—C2—C3—C4	179.4 (2)	C10—O2—C9—C7	−177.5 (2)
N1—C2—C3—C17	176.4 (2)	N5—C7—C9—O1	2.0 (4)
C16—C2—C3—C17	−3.4 (3)	C8—C7—C9—O1	−175.5 (3)
C2—C3—C4—C5	0.0 (3)	N5—C7—C9—O2	−179.2 (2)
C17—C3—C4—C5	−177.1 (2)	C8—C7—C9—O2	3.3 (3)
C2—C3—C4—C12	177.37 (18)	C9—O2—C10—C11	−87.7 (3)
C17—C3—C4—C12	0.3 (3)	C3—C4—C12—C13	−103.8 (3)
N1—C1—C5—C4	−2.2 (3)	C5—C4—C12—C13	73.5 (3)
N2—C1—C5—C4	179.51 (18)	C3—C4—C12—S1	70.8 (2)
N1—C1—C5—C6	177.1 (2)	C5—C4—C12—S1	−111.9 (2)
N2—C1—C5—C6	−1.2 (2)	C13—C12—S1—C15	−0.9 (2)
C3—C4—C5—C1	1.3 (3)	C4—C12—S1—C15	−176.20 (19)
C12—C4—C5—C1	−176.08 (18)	C4—C12—C13—C14	177.2 (2)
C3—C4—C5—C6	−177.6 (2)	S1—C12—C13—C14	2.2 (3)
C12—C4—C5—C6	5.0 (4)	C12—C13—C14—C15	−2.9 (3)
N2—N3—C6—N4	178.99 (19)	C13—C14—C15—S1	2.3 (4)
N2—N3—C6—C5	0.4 (3)	C12—S1—C15—C14	−0.8 (3)

### Hydrogen-bond geometry (Å, °)

**Table:**

*D*—H···*A*	*D*—H	H···*A*	*D*···*A*	*D*—H···*A*
N2—H1N2···N1^i^	0.89	2.07	2.941 (3)	165
N4—H1N4···O1	0.85	1.99	2.642 (3)	133
C13—H13A···N5^ii^	0.93	2.62	3.463 (3)	151

Symmetry codes: (i) −*x*, −*y*, −*z*; (ii) −*x*, −*y*+1, −*z*.
